# Supplementary material for: Alternative Splicing of TaHsfA2-7 Is Involved in the Improvement of Thermotolerance in Wheat
Source: Int J Mol Sci. 2023 Jan 5;24(2):1014. doi: 10.3390/ijms24021014 (PMC9861123; doi:10.3390/ijms24021014)
Supplement: Supplementary file 1 [file ijms-24-01014-s001.zip › ijms-2059092-supplementary.pdf]

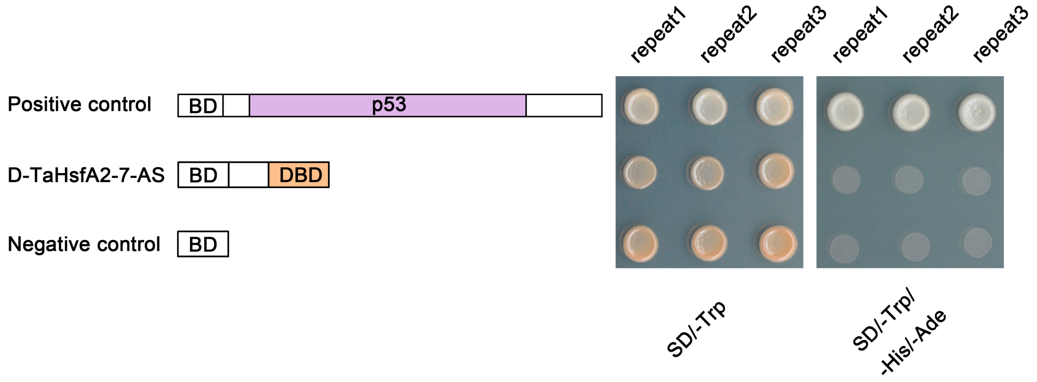

Figure S1. The effect of C-terminal hydrophobic sequence on the transcriptional activation of TaHsfA2-7-AS. Transcriptional activation analysis of D-TaHsfA2-7-AS in yeast. Fusion proteins of the GAL4 DNA-binding domain (BD) and D-TaHsfA2-7-AS were expressed in the yeast strain AH109. The transformed yeast cells were spotted onto SD/-Trp and SD/Trp-/His-/Ade- media. The plates were incubated at 30°C for 3 days. The pGBKT7 and pGBKT7-P53 vectors were used as negative and positive controls, respectively.

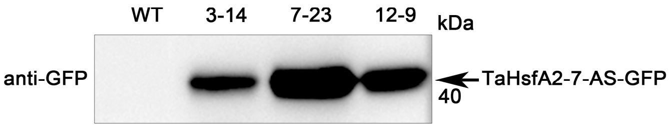

Figure S2. Protein detection of TaHsfA2-7-AS-GFP in transgenic Arabidopsis. The accumulation of TaHsfA2-7-AS-GFP in transgenic lines was detected with anti-GFP antibody (1: 5000, Sigma). Nontransgenic plant was used as control.

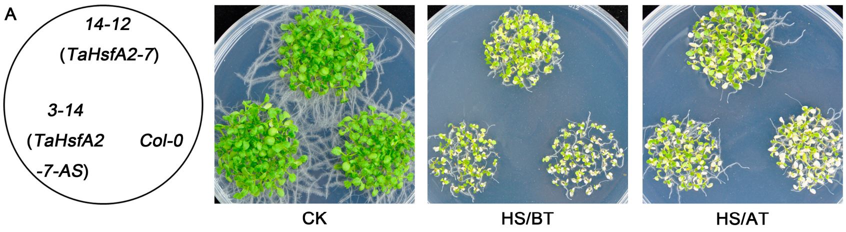

Figure S3. Thermotolerance assays of two splice variants in Arabidopsis. The phenotypes of WT and transgenic seedlings after basal thermotolerance (BT, 45 °C for 1 h, placed at 22 °C for 8 days) and acquired thermotolerance (AT, 37 °C for 1 h, incubated at 22 °C for 2 days, 46 °C for 1 h, placed at 22 °C for 8 days) treatment. The plants were photographed 8 d after different HS.

Table S1. Primers used in this study.

Assays	Gene	Forward primer (5'→3')	Reverse primer (5'→3')
RT-PCR	TaHsfA2-7-AS	ATGGACCGGGTGCTGCTG	CTACGCGTCGAAACATCT
RT-qPCR	TaHsfA2-7-AS	CTAGATGGATCAAGACACCGGCTGA	CATCTTCAGAAGCTGTCTCTGGC
	TaRP15	GCACACGTGCTTTGCAGATAAG	GCCCTCAAGCTCAACCATAACT
Transactivation analysis	TaHsfA2-7-AS	TCAGAGGAGGACCTGCATATGATGGACCGGGTGCTG	TCGACGGATCCCCGGGAATTCCTAGAACAAATAAGC
Subcellular localization	TaHsfA2-7-AS	GAGAACACGGGGGACTCTAGAATGGACCGGGTGCTG	GCCCTTGCTCACCATGGATCCGAACAAATAAGCAGG
Genetic transformation	TaHsfA2-7-AS	GAGAACACGGGGGACTCTAGAATGGACCGGGTGCTG	GCCCTTGCTCACCATGGATCCGAACAAATAAGCAGG
Protein purification	TaHsfA1	CGTGGATCCCCGGAATTCATGGACGGCGGGGTTGCTGCGGTGGC	ATGCGGCCGCTCGAGTCGACTCACCCTCTATGGTTGGATGACAGCA
Thermotolerance assays in yeast	TaHsfA2-7-AS	GGGAATATTAAGCTTGGTACCATGGACCGGGTGCTG	TACATGATGCGGCCCTCTAGACTAGAACAAATAAGC
Labeled probe	HSE1	GCGCCGCGCGGCCCAGAACCTTCCGCCCCGGCCCCCGTCC	GGACGGGGGCCGGGGCGGAAGGTTCTGGGCCGCGCGGCGC
	HSE2	CGGCCCCGGCGTCCAGAAGCTTCCGAGCCGGCCGGTCACG	GGACGGGGGCCGGGGCGGAAGGTTCTGGGCCGCGCGGCGC
	HSE3	AAGTGATATTTGAGAGAAAATTCCTTATTTGACACTATCTTAA	TTAAGATAGTGTCAAATAAGGAATTTTCTCTCAAATATCACTT
Labelled mutant probe	HSE1	GCGCCGCGCGGCCCAAAAAATTACGCCCCGGCCCCCGTCC	GGACGGGGGCCGGGGCGTAATTTTTTGGGCCGCGCGGCGC
	HSE2	CGGCCCCGGCGTCCAAAAAATTACGAGCCGGCCGGTCACG	CGTGACCGGCCGGCTCGTAATTTTTTGGACGCCGGGGCCG
	HSE3	AAGTGATATTTGAGAAAAAATTACTTATTTGACACTATCTTAA	TTAAGATAGTGTCAAATAAGTAATTTTTTCTCAAATATCACTT
